# Persistent low serum zinc is associated with recurrent sepsis in critically ill patients - A pilot study

**DOI:** 10.1371/journal.pone.0176069

**Published:** 2017-05-04

**Authors:** Janine Hoeger, Tim-Philipp Simon, Thorben Beeker, Gernot Marx, Hajo Haase, Tobias Schuerholz

**Affiliations:** 1 Department of Intensive Care and Intermediate Care, University Hospital Aachen, RWTH Aachen University, Aachen, Germany; 2 Department of Food Chemistry and Toxicology, Berlin Institute of Technology, Berlin, Germany; 3 Department of Anesthesiology and Intensive Care, University of Rostock, Rostock, Germany; Azienda Ospedaliero Universitaria Careggi, ITALY

## Abstract

Zinc is an essential trace element for both pathogens and hosts. Hypozincemia is a well known phenomenon in sepsis patients and represents the innate immune systems attempt to deprive pathogens of zinc. However little is known about course, restitution and prognostic value of serum zinc levels in sepsis patients. We performed a prospective observational single-center study set in a tertiary care university hospital intensive care unit. Serum zinc levels were singularly measured of healthy controls and sequentially of surgical sepsis patients and surgical patients over a 8-day period. Throughout the study period, we report significantly decreased serum zinc levels in surgical and surgical sepsis patients compared to healthy controls. Lower serum zinc levels in surgical sepsis patients were associated with a higher susceptibility to a recurrent sepsis episode. Furthermore, surgical sepsis patients with a higher number of organ dysfunctions and increased in-hospital mortality at day 28 and 90 showed lower serum zinc levels at admission. We report serum zinc levels as a promising biomarker in the diagnosis and evaluation of sepsis patients. However, it is still unclear whether these findings are caused by an over-amplified redistribution of zinc during acute-phase response, or whether the critically ill patients were zinc deficient before sepsis.

## Introduction

Sepsis is a severe condition and the most common cause of death in critically ill patients [1]. As implemented by the Surviving Sepsis Campaign [2], time to adequate therapy is the major factor in the acute management of this condition. Mortality rates of sepsis patients increase for each and every hour without antimicrobial therapy [3]. Besides the high mortality in the acute phase of sepsis there is growing evidence that survivors of sepsis are more susceptible to infections, resulting in an increased re-hospitalization rate, higher morbidity and consecutively higher mortality [4].

Zinc is an essential micronutrient, and plays an important role in a myriad of cellular processes such as protein synthesis, DNA repair and cytoprotection. Hypozincemia is a well observed phenomenon in sepsis patients [5]. In acute phase response zinc is redistributed to the liver and other organs, leading to a transient decrease in serum zinc levels [6]. This mechanism is an important feature of host immune defense to limit availability of essential micronutrients to pathogens and is also known as nutritional immunity [7]. Wong et al. reported perturbed genome level expression in pediatric septic shock non-survivors, mainly affecting zinc homeostasis and leading to lower serum zinc levels in non-survivors [8]. Furthermore, zinc deficiency compromises immune function [9]. In a murine model of polymicrobial sepsis zinc deficiency increased mortality rate and burden of disease [10]. Especially in elderly patients, persistent zinc deficiency is a common condition and associated with a higher susceptibility to infections [11], due to an impaired immune response [12].

We hypothesized that transient hypozincemia in sepsis patients might be associated with a higher susceptibility to sepsis. To determine the duration of hypozincemia, we measured sequential serum zinc levels in critically ill patients.

## Materials and methods

### Study population

All patients were recruited consecutively in the Department of Intensive Care and Intermediate Care of the University Hospital Aachen between September 2012 and March 2014. In total 44 surgical sepsis patients, 18 surgical patients and 20 healthy controls were enrolled. Patients with sepsis were enrolled within 36 hours after onset of sepsis defined by at least 2 SIRS criteria and suspected or proven infection. To determine serum zinc levels at the very onset of sepsis we subdivided patients, who were enrolled within 18 hours after onset of sepsis and declared this time point as TA (Time of admission). All consecutive samples were drawn according to the protocol. All surgical patients were admitted immediately to ICU after surgery. The study protocol was reviewed and approved by an institutional review board (Ethics Committee of the medical faculty, Rheinisch-Westfälische Technische Hochschule, RWTH Aachen, Pauwelsstrasse 30, 52074 Aachen, chairman Professor Dr. med. G. Schmalzing (Register, EC Nr. 032/12)). Informed consent was obtained before inclusion and any study related measure by patients or their legal representatives.

### Sample collection

In accordance to the protocol blood samples of septic and surgical patients were drawn from already inserted intravascular catheter after recruitment within 18 hours after onset of sepsis (TA), or at day 1 (T1), day 2 (T2) and day 3 (T3). Samples were taken only during ICU stay. Healthy controls consented to a single peripheral blood sample. Serum samples were allowed to clot at room temperature for 30 minutes and consecutively centrifuged at 2000g for 10 minutes. Remaining samples were transferred to a biobank after additional consent was obtained from the patient, or their legal representative.

### Atomic absorption spectrometry (AAS)

All reagents for atomic absorption measurements were of appropriate quality for trace element analysis (TraceSelect, Fluka, Germany). Serum samples were diluted 1:5 in ultrapure water containing 0.2% (v/v) HNO_3_ and were analyzed by flame AAS on a Perkin Elmer AAnalyst 800 instrument. Acetylene flow was set to 2.0 l/min and oxidant (air) to 17.0 l/min. A reference serum and serum samples spiked with defined concentrations of the respective ions were routinely analyzed to ensure reliable quantification [13].

### Data collection and definitions

All relevant data were extracted from medical records and electronic bedside flow charts (IntelliSpace Critical Care and Anesthesia (ICCA); Philips Healthcare, Andover, Massachusetts, USA). C-reactive protein (CRP), pro-calcitonin (PCT), Lactate-Dehydrogenase (LDH), serum-lactate and Sequential Organ Failure Assessment (SOFA) Score laboratory parameters were routinely determined.

Sepsis, severe sepsis and septic shock were defined in accordance with the diagnostic criteria of the American College of Chest Physicians/ Society of Critical Care Consensus Conference [14, 15]. In-hospital mortality was determined from the discharge status and medical records.

A recurrent sepsis episode was defined as either a different source of sepsis or a complete remission of infection and consecutive relapse within 28 days after enrolment. Patients who died in the course of the initial septic episode were excluded from this subanalysis.

### Statistical analysis

Data analysis was performed using IBM SPSS Statistics 22.1 and GraphPad Prism Version 5.01 (Graphpad Software, San Diego, CA, USA). In all calculations a p <0.05 was considered statistically significant. After verifying normal distribution the following analysis was performed: Univariate analysis was performed using one-way analysis of variances (ANOVA) followed by either Dunnett’s or Tukey’s post hoc test. When only two groups were compared we used an unpaired t-test followed by Bonferroni-Holm procedure for multiple comparison.

## Results

In this study 44 surgical sepsis patients, 18 surgical patients and 20 healthy controls were enrolled. Demographic characteristics of the patients are depicted in Table 1. Mean age and gender distribution did not differ significantly between the groups. Mortality and SOFA Score were highest in septic shock patients (Table 1).

**Table 1:** Demographic characteristics of the study population.

Mean ± SEM or (%) per group	Healthy Controls (n = 20)	Surgical Controls (n = 18)	Sepsis (n = 11)	Severe Sepsis (n = 7)	Septic Shock (n = 26)
**Age [years]**	60.70±3.64	66.44±2.37	68.00±3.71	64.29±5.19	68.23±2.55
**BMI [kg/m**^**2**^**]**		29.82±2.21	26.65±1.16	34.11±5.84	26.25±1.00
**APACHE II TA**		8.06_a_±0.86	10.27_a_±1.62	12.14±1.37	18.38_a_±1,24
**SOFA TA**		3.50_a_±0.61	3.50_a_±0.60	5.20±0.58	8.80a±0.69
**Sex [Male]**	55.0%	61.1%	63.6%	57.1%	46.2%
**Mortality 28 [days]**	0.0%	0.0%	0.0%	14.3%	26.9%
**Mortality 90 [days]**	0.0%	5.6%	9.1%	28.6%	30.8%

Significant differences between the groups are indicated by a (p<0.05)

### Serum zinc in the diagnosis of sepsis and association with sepsis severity

**Fig 1:**
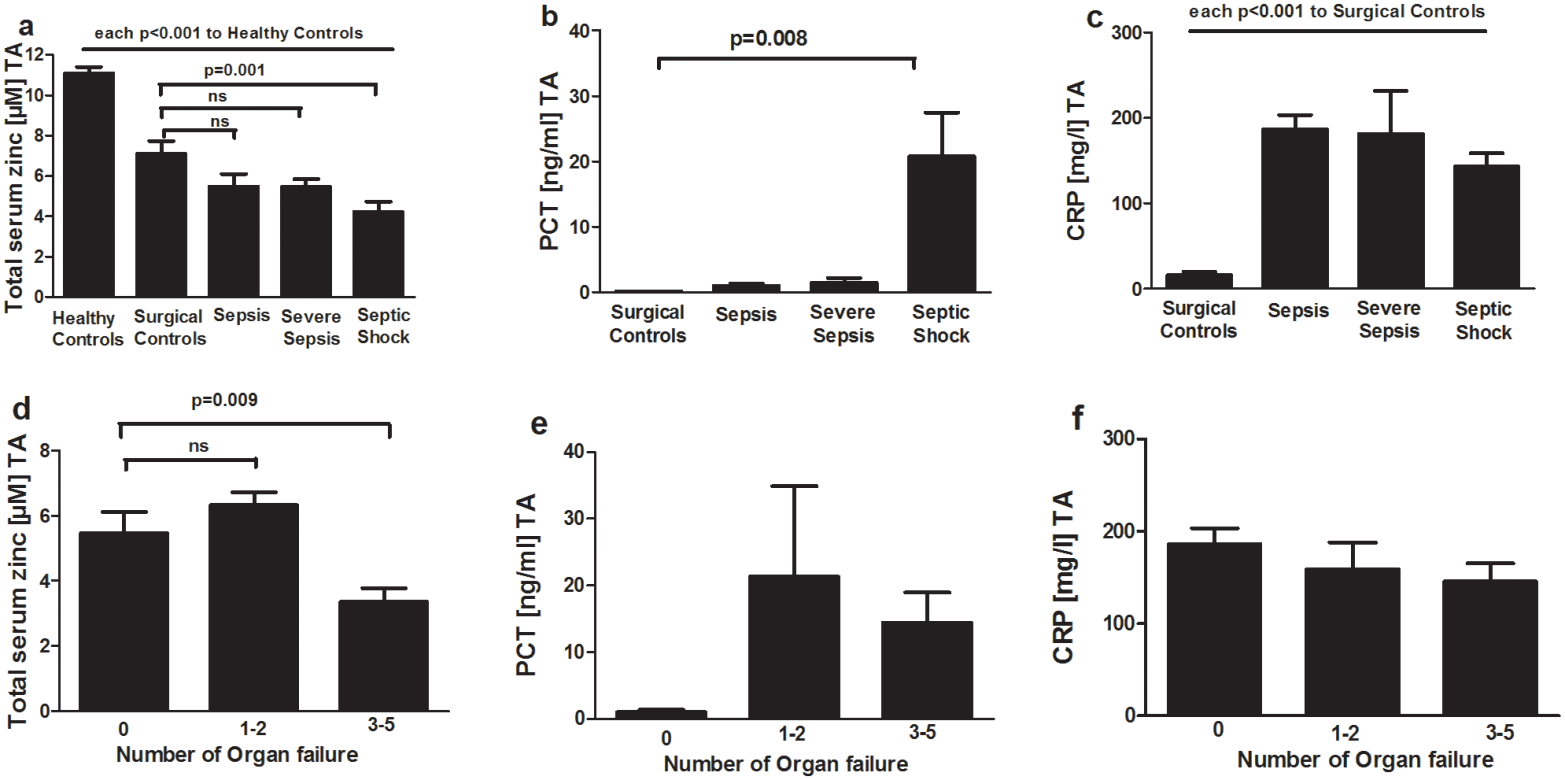
Total serum zinc, Procalcitonin (PCT) and C-reactive Protein (CRP) levels at admission (TA) divided by groups (a-c) or number of organ dysfunctions (d-f) among sepsis patients. Data are shown as mean + SEM and analyzed by one-way ANOVA followed by Dunnett’s post hoc test.

Serum zinc levels at admission were significantly decreased in surgical and septic patients in comparison to healthy controls (11.10 ± 0.30 μM zinc healthy controls vs. 7.09 ± 0.65 μM zinc surgical patients, p<0.001). Though, only patients presenting with septic shock had significantly lower serum zinc concentrations compared to surgical patients (4.21 ± 0.51 μM septic shock zinc, p = 0.001 vs.7.09 ± 0.65 μM zinc surgical patients) (Fig 1a). Similar to these results PCT serum levels at admission differed only significantly between surgical patients and septic shock patients (0.12 ± 0.03ng/ml PCT surgical patients vs. 20.77 ± 6.67 ng/ml PCT septic shock) (Fig 1b). In contrast, CRP levels at admission differed significantly between septic patients and surgical patients (each p<0.001 compared to surgical patients) (Fig 1c). The number of organ dysfunction among sepsis patients was assessed according to the criteria of the American College of Chest Physicians/ Society of Critical Care Consensus Conference [14, 15]. Serum zinc levels at admission were significantly lower in surgical sepsis patient with multiple organ dysfunctions compared to surgical sepsis patients without organ dysfunctions(3 to 5 organ dysfunctions: 3.35 ± 0.42 μM zinc vs. no organ dysfunction: 5.46 ± 0.65 μM zinc) (Fig 1d).

### Patients with low serum zinc levels are at risk to develop a recurrent sepsis episode

**Fig 2:**
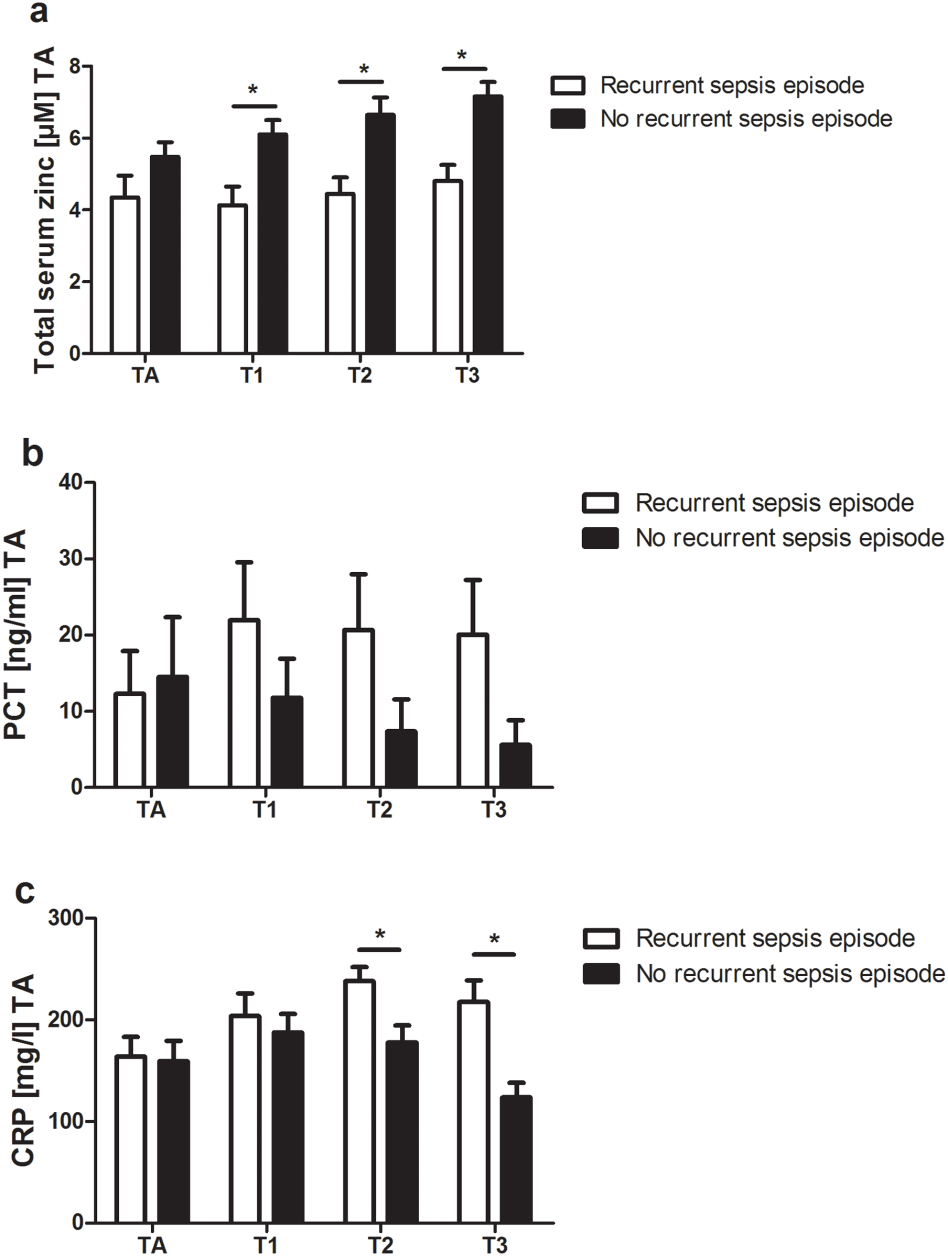
Development of a recurrent sepsis episode in sepsis patients within 28 days after enrolment. Sepsis patients, who died within the first septic episode, were excluded from this subset analysis. Sequentially measured a) Total serum zinc, b) Procalcitonin (PCT) and c) C-reactive protein (CRP) levels at admission (TA) are shown as mean + SEM and analyzed by unpaired t-test followed by Bonferroni-Holm procedure for multiple comparison. Significant differences are indicated by * (= p ≤ 0.05).

A recurrent sepsis episode was defined as described above. Patients who died within the first septic episode were excluded from this subset analysis. Once again the two analyzed groups showed a similar severity of diseases according to the SOFA score at admission (Fig 2; Table 2).

**Table 2:** Characteristics of sepsis patients subdivided by in-hospital mortality, occurrence of a recurrent sepsis episode within 28days and source of sepsis.

Mean ±SEM	Outcome 28 days		Focus		Recurrent sepsis episode within 28 days	
	Survivors (n = 36)	Non-Survivors (n = 8)	Pulmo (n = 20)	Abdomen (n = 17)	Recurrent sepsis episode (n = 19)	No recurrent sepsis episode (n = 20)
**SOFA TA**	6.29 ± 0.57	10.80* ± 1.74	6.86 ± 0.92	6.69 ± 0.94	6.92 ± 0.51	5.81 ± 0.90
**APACHE II TA**	13.78 ± 0.99	22.50 *± 1.95	14.70 ± 1.25	14.18 ± 1.91	17.47 ± 1.57	12.00* ± 1.15
**Serum zinc levels [μM]** **zinc TA** **zinc T1** **zinc T2** **zinc T3**	5.09 ± 0.35 5.22 ± 0.34 5.56 ± 0.39 5.94 ± 0.40	2.08* ±0.39 4.18* ± 1.21 5.08 ± 1.03 5.48 ± 0.86	5.61 ± 0.47 6.02 ± 0.49 6.73 ± 0.49 6.76 ± 0.41	4.04 ± 0.47 4.15* ± 0.49 4.52 *± 0.44 5.14 *± 0.65	4.35 ± 0.61 4.13 ± 0.52 4.45 ± 0.45 4.81 ± 0.45	5.48 ± 0.41 6.11 *± 0.40 6.65 *± 0.49 7.16 *± 0.40
**PCT TA [mg/ml]**	13.80 ± 5.09	8.89 ± 3.63	19.83 ± 9.35	7.76 ± 3.37	12.28 ± 5.63	14.52 ± 7.82
**CRP TA [mg/l]**	163.90 ± 14.15	130.25 ± 24.45	136.36 ± 19.83	183.35 ± 18.10	163.81 ± 19.61	159.36 ± 20.05
**LDH TA [U/l]**	399.50 ± 104.17	382.20 ± 176.14	524.14 ± 202.53	316.94 ± 62.70	317.46 ± 45.75	456.13 ± 179.86
**Lactate [mmol/l]**	2.17 ± 0.36	10.86* ± 4.01	1.86 ± 0.38	4.13 ± 1.21	2.22 ± 0.57	2.07 ± 0.44
**Hours of ventilator support within 28days**	130.42 ± 38.32	158.5 ± 82.2	68.0 ± 24.4	232.12* ± 78.5	282.26 ± 65.52	21.2* ± 10.3
**Hours of vasopressor support within 28 days**	85.4 ± 24.90	161.6 ± 62.7	49.47 ± 15.1	160.1 ± 53.8	190.13 ± 46.32	25.4 ± 7.4
**Max. Norepinephrine [μg/kg/min]**	0.25 ± 0.08	0.77* ± 0.30	0.18 ± 0.07	0.42 ± 0.16	0.44 ± 0.17	0.10 ± 0.03

Significant differences between the groups are indicated by * (p<0.05).

Total serum zinc levels of patients who developed a new septic episode were significantly lower at day 1, 2 and 3. Furthermore, total serum zinc levels did not increase significantly over time but remained at a low level.

In patients without a recurrent sepsis episode, total serum zinc levels increased over the study period. In line with these findings PCT and CRP serum levels in patients who developed a recurrent sepsis episode remained higher, and did not decrease over time. However, there was a significant difference between the two groups in CRP serum levels at day 2 and 3 only.

### Low serum zinc levels at admission are associated with worse outcome in sepsis patients

Total serum zinc levels at admission were significantly lower in non-survivors at day 28 and 90 (day 28 survivors 5.09 ± 0.35 μM zinc vs. 2.08 ± 0.39 μM zinc in non-survivors; day 90 survivors 5.24 ± 0.35 μM zinc vs. 2.47 ± 0.57 μM zinc in non-survivors). Therefore, low serum zinc levels were associated with higher in-hospital mortality.

**Fig 3:**
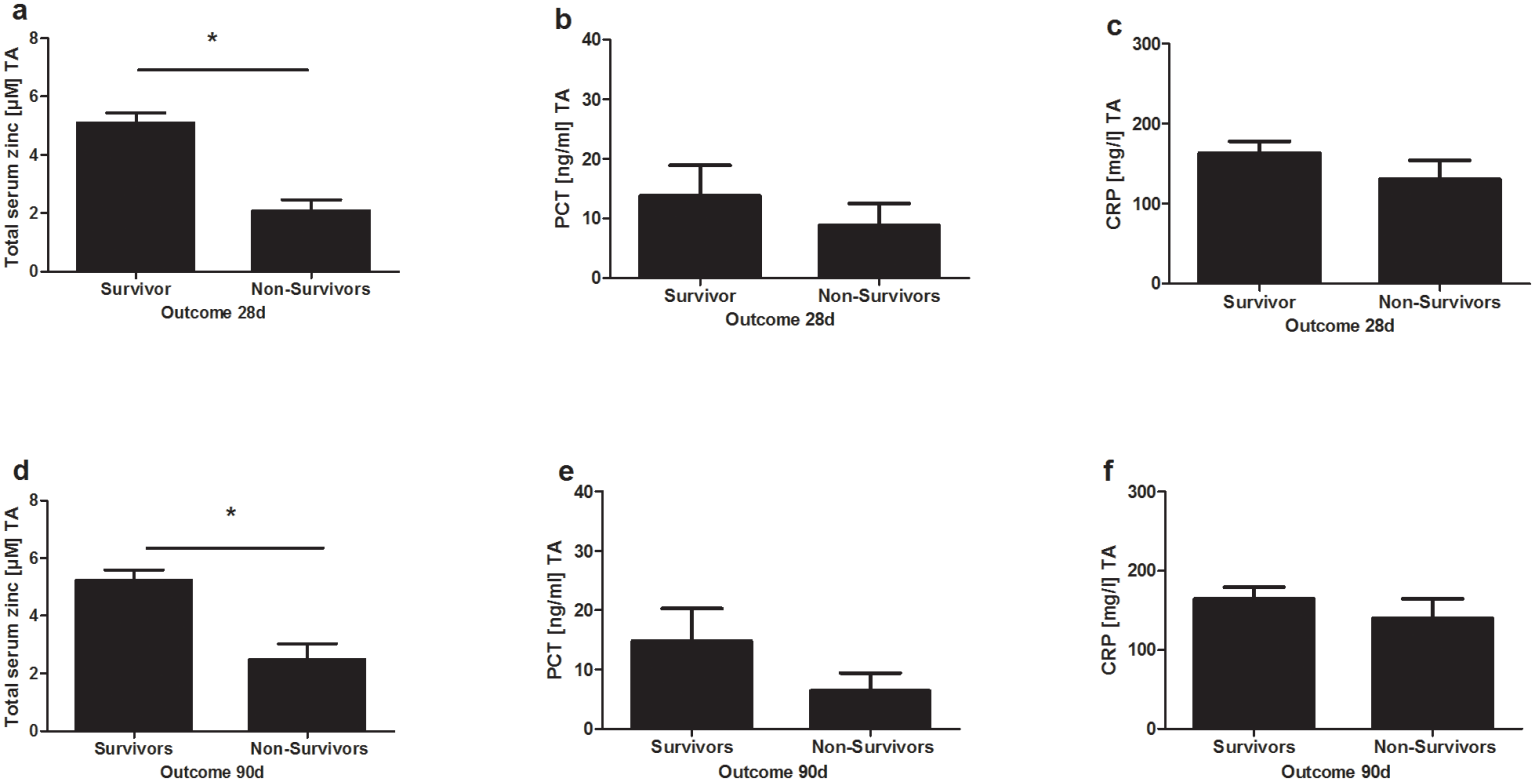
In- hospital mortality of sepsis patients after 28 (a-c) and 90 days (d-f) of enrollment. Total serum zinc, Procalcitonin (PCT) and C-reactive protein (CRP) levels at admission (TA) are shown as mean ± SEM in survivors and non-survivors of sepsis. Significant differences are indicated by * (= p ≤ 0.05).

Non-survivors suffered from a higher disease severity compared to survivors, indicated by significantly higher maximal vasopressor concentrations at admission, serum lactate and SOFA and APACHE II Scores. However, PCT and CRP serum levels did not predict outcome, though PCT levels tended to be lower in non-survivors (Fig 3; Table 2).

## Discussion

Zinc is essential for immune cell function on various levels and an indispensable second messenger in a multitude of signal pathways [16, 17]. Zinc deficiency impairs immune function and is associated with a higher susceptibility to infections. In addition to an impaired immune response and inefficient bacterial clearance, zinc deficiency enhances the inflammatory response. The over-amplification of the inflammatory response leads to increased organ damage and consecutively higher mortality [18]. Besecker et al. observed higher cytokine concentrations and an increased severity of sepsis in sepsis patients with lower serum zinc levels [19]. Furthermore, Mertens et al. found that Zinc and selenium concentrations, in particular in the patients with sepsis, were reduced in plasma of patients with increased oxidative stress and inflammatory biomarkers on intensive care unit and concluded that “oxidative stress as a result of suboptimal selenium and zinc concentrations might contribute to damage of key proteins”[20].

We report persistent lower serum zinc levels in patients suffering a recurrent sepsis episode. These results might indicate a need for zinc supplementation in sepsis patients. So far only prophylactic zinc supplementation has been shown to reduce mortality in animal models [10, 21], most likely by improving the immune status before sepsis. All attempts to supplement zinc in the course of sepsis were disappointing in adults. Zinc supplementation in the course of infection might abrogate the limitation of zinc to pathogens, which in turn could lead to increased bacterial growth.

We recently reported a reduced zinc binding capacity in a porcine model of sepsis when zinc was added to the serum samples due to the downregulation of albumin, the major zinc binding protein in serum [13]. In terms of zinc supplementation the decrease in zinc binding capacity results in a narrow therapeutic window, rendering potentially harmless doses of zinc into toxic doses. Therefore, further studies are warranted to evaluate not only the beneficial effects, but also the safety of a potential therapeutic zinc supplementation in sepsis patients.

The measurement of serum zinc levels might be a useful parameter in the diagnosis and evaluation of sepsis especially in combination with the established biomarkers. To date there is no single infallible biomarker in the diagnostic of sepsis but rather a bouquet of parameters. PCT is the most established biomarker in the diagnosis of sepsis and discrimination between infectious and non-infectious inflammation [22]. Nevertheless, the diagnostic value of PCT underlies certain restrictions especially in patients with renal failure. Therefore, we were not able to show a superiority of PCT in the diagnosis of infection due to our small number of patients. CRP on the other hand provides a slow kinetic profile, which explains the superior discrimination between surgical and sepsis patients, since blood samples of surgical patients were drawn immediately after admission to Intensive Care Unit (ICU). In our study, we report an association of multiple organ dysfunction and increased mortality with lower serum zinc levels at admission. CRP and PCT serum levels, however, were associated neither with the number of organ dysfunctions nor with outcome.

Additionally, we observed significantly lower serum zinc levels in patients with abdominal source of sepsis in comparison to patients with pulmonary sepsis for the observed period of time (Table 2). The groups showed comparable SOFA scores, however patients with abdominal sepsis had higher vasopressor support and were significantly longer depended on ventilator support (Table 2). The difference in serum zinc levels might be therefore derived by a higher burden of disease in patients with abdominal source of sepsis in our study.

Further studies are warranted to determine the validity of serum zinc levels in the prediction of sepsis severity in comparison to the established score systems and to investigate zinc homeostasis stratified by the source of infection.

Van Zanten et al sequentially measured serum zinc levels at baseline, day 4 and day 8 in medical, surgical and trauma patients receiving mechanical ventilation for more than 72 hours [23]. This study was a randomized controlled trial exploring the effects of immune-modulating high protein or isocaloric high protein enteral nutrition in critically ill patients and was abrogated due to negative outcome in the intervention group. Van Zanten et al. reported no differences in baseline zinc plasma levels in their study group. The supplemented immune-modulating high protein nutrition contained not only zinc, but glutamine, fish oil and other antioxidants.

Based on these findings Hofman et al. performed a post hoc safety analysis and reported a significant association between baseline plasma concentration of zinc and 6-month mortality in the medical critically ill patients, which is in accordance to our findings. The safety analysis indicated an inverse association of low serum levels of zinc and the development of organ dysfunctions [24]. Furthermore, Hofman et al. demonstrated a statistically significant negative association between changes in zinc from baseline to day 8 and 6-month mortality in the non-medical patients. However, this association between serum zinc levels and mortality is lost in non-medical critically ill patients and in the general patient population (24). This fact, in turn, may indicate an inverse association between low zinc levels and development of organ dysfunction, but does not necessarily support a beneficial effect of zinc supplementation in non-medical critically patients. Our study has some limitations. To the best of our knowledge, our study is the first observational study evaluating sequential serum zinc levels in critically ill surgical patients with sepsis. In this context we conducted a single-center exploratory pilot study, which might limit the generalization of our results. Blood samples of surgical patients were drawn immediately after admission to ICU, resulting in an earlier stage of inflammation in comparison to sepsis patients. Furthermore, we were not able to assess the zinc status of sepsis patients before infection, and did not evaluate the possible effect of zinc supplementation in sepsis patients. We concede that present findings should be considered within the limits of an exploratory pilot study, which does not prove a causal relationship.

## Conclusion

Surgical sepsis patients with multiple organ dysfunctions and increased in-hospital mortality showed significantly lower serum zinc levels. We report serum zinc levels as a promising biomarker in the diagnosis and evaluation of sepsis patients. However, it is still unclear whether these findings are caused by an over-amplified redistribution of zinc during acute-phase response, or whether the critically ill patients were zinc deficient before sepsis.

## Supporting information

S1 Data Primary data sheet. (XLS)
